# Cardiac vagal dysfunction and myocardial injury after non-cardiac surgery: a planned secondary analysis of the measurement of Exercise Tolerance before surgery study

**DOI:** 10.1016/j.bja.2018.10.060

**Published:** 2018-12-17

**Authors:** T.E.F. Abbott, R.M. Pearse, B.H. Cuthbertson, D.N. Wijeysundera, G.L. Ackland

**Affiliations:** 1 William Harvey Research Institute, Queen Mary University of London, London, UK; 2 University College London Hospital, London, UK; 3 Barts Health NHS Trust, London, UK; 4 Department of Critical Care Medicine, Sunnybrook Health Sciences Centre, Toronto, ON, Canada; 5 University of Toronto, Toronto, ON, Canada; 6 Li Ka Shing Knowledge Institute, St. Michael's Hospital, Toronto, ON, Canada; 7 Toronto General Hospital, Toronto, ON, Canada

**Keywords:** cardiopulmonary exercise testing, heart rate, myocardial injury after non-cardiac surgery, B-type natriuretic peptide, surgery, troponin, vagal function

## Abstract

**Background:**

The aetiology of perioperative myocardial injury is poorly understood and not clearly linked to pre-existing cardiovascular disease. We hypothesised that loss of cardioprotective vagal tone [defined by impaired heart rate recovery ≤12 beats min^−1^ (HRR ≤12) 1 min after cessation of preoperative cardiopulmonary exercise testing] was associated with perioperative myocardial injury.

**Methods:**

We conducted a pre-defined, secondary analysis of a multi-centre prospective cohort study of preoperative cardiopulmonary exercise testing. Participants were aged ≥40 yr undergoing non-cardiac surgery. The exposure was impaired HRR (HRR≤12). The primary outcome was postoperative myocardial injury, defined by serum troponin concentration within 72 h after surgery. The analysis accounted for established markers of cardiac risk [Revised Cardiac Risk Index (RCRI), N-terminal pro-brain natriuretic peptide (NT pro-BNP)].

**Results:**

A total of 1326 participants were included [mean age (standard deviation), 64 (10) yr], of whom 816 (61.5%) were male. HRR≤12 occurred in 548 patients (41.3%). Myocardial injury was more frequent amongst patients with HRR≤12 [85/548 (15.5%) *vs* HRR>12: 83/778 (10.7%); odds ratio (OR), 1.50 (1.08–2.08); *P*=0.016, adjusted for RCRI). HRR declined progressively in patients with increasing numbers of RCRI factors. Patients with ≥3 RCRI factors were more likely to have HRR≤12 [26/36 (72.2%) *vs* 0 factors: 167/419 (39.9%); OR, 3.92 (1.84–8.34); *P*<0.001]. NT pro-BNP greater than a standard prognostic threshold (>300 pg ml^−1^) was more frequent in patients with HRR≤12 [96/529 (18.1%) *vs* HRR>12 59/745 (7.9%); OR, 2.58 (1.82–3.64); *P*<0.001].

**Conclusions:**

Impaired HRR is associated with an increased risk of perioperative cardiac injury. These data suggest a mechanistic role for cardiac vagal dysfunction in promoting perioperative myocardial injury.

**Editor's key points**

• The hypothesis of this study was that cardiac vagal dysfunction, manifesting as impaired heart rate deceleration after exercise (defined as heart rate recovery ≤12 beats min^−1^), is associated with perioperative myocardial injury.
• To test this, investigators conducted a planned sub-group analysis of patients enrolled in an international, prospective, multi-centre cohort study.
• Patients with an increased preoperative Lee's Revised Cardiac Risk Index, and those with an elevation of N-terminal pro B-type natriuretic peptide (a counter-regulatory hormone released by the ventricle in the setting of cardiac dysfunction), had impaired heart rate deceleration after cardiopulmonary exercise testing.
• As hypothesised, impaired heart rate deceleration after exercise was independently associated with myocardial injury, based on troponin elevation, after non-cardiac surgery.
• Cardiac vagal dysfunction could increase perioperative myocardial oxygen requirements, and thus might be an important mechanistic contributor to perioperative myocardial injury.

The majority of the estimated 300 million surgical patients each year undergo non-cardiac procedures.^1^ Around 20% of non-cardiac surgical patients sustain perioperative myocardial injury,^2^, ^3^ which is usually asymptomatic yet strongly associated with hospital readmission^4^ and mortality.^5^, ^6^, ^7^, ^8^, ^9^ Myocardial injury is more common in patients with established cardiovascular risk factors, as estimated using the Revised Cardiac Risk Index (RCRI).^6^, ^10^, ^11^, ^12^ However, conventional treatments for myocardial infarction do not reduce myocardial injury, cardiovascular death, or both after non-cardiac surgery.^13^, ^14^, ^15^ Moreover, objective measures of atherosclerosis using computed tomography coronary angiogram correlate poorly with the risk of perioperative myocardial injury and do not increase the predictive utility of the RCRI.^16^

Resting heart rate is independently associated with cardiovascular morbidity and mortality, both in the general population^17^, ^18^ and in patients undergoing non-cardiac surgery.^6^, ^19^ Cardiac vagal activity is the major autonomic determinant of resting heart rate.^20^, ^21^ In pathologic settings, the loss of cardiac vagal activity exacerbates myocardial cellular injury after acute inflammation, haemorrhage, and ischaemia.^22^, ^23^, ^24^, ^25^ Parasympathetic dysfunction, as reflected by delayed heart rate recovery (HRR) after graded exercise, is common among people with cardiometabolic risk factors that comprise the RCRI.^16^, ^26^ Parasympathetic dysfunction could therefore promote myocardial injury, exacerbate myocardial injury, or both through several relevant pathophysiological mechanisms characterised by acute inflammation, tissue oxygen supply–demand imbalance, arterial hypotension, or both. Loss of cardioprotective mechanisms to counteract such cardiovascular challenges may result in myocardial injury. Taken together, it is plausible that established cardiac vagal dysfunction may be an unrecognised factor in promoting perioperative myocardial injury.

The Measurement of Exercise Tolerance before Surgery (METS) study found no relationship between objective markers of exercise capacity measured using cardiopulmonary exercise testing (CPET; peak oxygen consumption and anaerobic threshold) and perioperative myocardial injury.^27^ In the same large prospective multi-centre cohort study, we prospectively tested the hypothesis that cardiac vagal dysfunction, as defined by impaired HRR, was associated with increased risk of myocardial injury after non-cardiac surgery. HRR, which was measured after preoperative CPET,^28^ is an established measure of cardiac vagal tone that is associated with all-cause mortality, independent of other exercise test parameters.^29^, ^30^ We further tested this hypothesis by exploring whether impaired HRR was associated with established preoperative risk factors for postoperative cardiovascular complications, on the basis that loss of cardioprotective vagal activity (as reflected by lower HRR) provides a plausible unifying mechanism linking clinical and biochemical predictive indicators with perioperative myocardial injury.

## Methods

### Study design and setting

This was a pre-defined secondary analysis of the METS study, an international prospective observational cohort study of preoperative assessment before non-cardiac surgery at 25 hospitals in Canada, UK, Australia, and New Zealand.^27^ The study protocol and methods were published previously.^31^ The study received research ethics approval before participant recruitment started and was conducted in accordance with the principles of the Declaration of Helsinki and the Research Governance Framework.

### Participants

Participants were 40 yr of age or older, undergoing elective non-cardiac surgery under general anaesthesia, regional anaesthesia, or both with a planned overnight stay in hospital, and with at least one of the following perioperative risk factors: intermediate or high-risk surgery, coronary artery disease, heart failure, cerebrovascular disease, diabetes mellitus, preoperative renal insufficiency, peripheral arterial disease, hypertension, a history of tobacco smoking within the previous year, or aged 70 yr or older. The exclusion criteria were: planned procedure using only endovascular technique, use of CPET for risk stratification as part of routine care, insufficient time for CPET before surgery, presence of an implantable cardioverter–defibrillator, known or suspected pregnancy, previous enrolment in the study, severe hypertension (>180/100 mm Hg), active cardiac conditions, or other contraindications precluding CPET.^31^, ^32^ Participants gave written informed consent to take part before surgery.

### Study conduct and data collection

A detailed and standardised dataset was collected before surgery, during the hospital stay, and at 30 days and 1 yr after surgery. Researchers collected data directly from participants and their medical record. Each participant underwent preoperative CPET. Blood was sampled before surgery and on the first, second, and third days after surgery, as long as the participant remained in hospital. In Canada, Australia, and New Zealand, serum cardiac troponin (either I or T isoforms) concentration was measured in preoperative and postoperative samples at local hospital laboratories, according to local policy. In the UK, serum cardiac troponin was measured in preoperative and postoperative samples at a single central laboratory. A summary of the troponin assays used at each centre is summarised in Supplementary Table S1. N-terminal pro-hormone of brain natriuretic peptide (NT pro-BNP) concentration, which has been shown to predict perioperative cardiac events, was measured in all preoperative samples at a single central laboratory.^33^ Electrocardiograms were performed before surgery and on the first, second, and third days after surgery.

### Cardiopulmonary exercise testing

Participants underwent preoperative symptom-limited CPET using a standardised incremental ramp protocol using electromagnetically braked cycle ergometers, as described and published previously.^27^, ^31^ HRR during the first minute of the recovery period was calculated as the difference between heart rate at the end of the incremental exercise and heart rate after 1 min of the recovery period. Clinicians, patients, and outcome adjudicators were blinded to the results of CPET, except where there was a safety concern according to pre-defined criteria.^31^

### Exposures and outcomes

The exposure of interest was impaired HRR, defined as reduction in heart rate of ≤12 beats min^−1^ during the first minute after the end of preoperative CPET. This threshold is prognostically associated with subsequent cardiovascular morbidity in the general^29^ and surgical populations.^34^ The primary outcome measure was myocardial injury, defined as blood troponin T or I concentration greater than the limit of the reference range (99th centile) for each assay, within 72 h after surgery. Troponin assays differed between participating hospitals and are listed in Supplementary Table S1. Pre-defined explanatory variables that may confound an association between impaired HRR and myocardial injury are commonly used preoperative cardiovascular risk indicators, namely, NT pro-BNP concentration and RCRI, which are both prognostically associated with postoperative myocardial infarction.^33^, ^35^ We used a threshold of >300 pg ml^−1^ for preoperative NT pro-BNP concentration which appears to predict postoperative cardiovascular events in surgical patients.^33^

### Statistical analysis

We used STATA version 14 (STATACorp LP, College Station, TX, USA) to analyse the data. The small number of participants without a record of HRR were excluded. We ranked the sample by HRR at 1 min after the end of incremental exercise and dichotomised it according to a threshold of ≤12 beats min^−1^. We presented baseline characteristics for the whole cohort and stratified by HRR. Normally distributed data were expressed as mean (standard deviation, sd), and non-normally distributed data were expressed as median (inter-quartile range, IQR). Binary data were expressed as percentages. We performed a complete case analysis. First, we used univariable logistic regression analysis to measure the unadjusted association between impaired HRR and myocardial injury. Second, using a previously published method for stratifying patients at risk of perioperative myocardial injury, we divided the cohort into three groups according to RCRI [low-risk (RCRI 0 points), intermediate-risk (RCRI 1–2 points), or higher risk^35^ (RCRI 3–6 points)], which represents multiple cardiovascular risk factors known to be associated with perioperative myocardial injury.^6^, ^8^, ^10^, ^11^, ^35^, ^36^, ^37^ We constructed a multivariable logistic regression model to determine the association between impaired HRR and myocardial injury, after adjustment for RCRI, where the low-risk group was considered the reference category. Third, we repeated the multivariable logistic regression model, adjusted for component cardiovascular risk factors of the RCRI, including: coronary artery disease, heart failure, diabetes mellitus requiring insulin therapy, and preoperative renal insufficiency (creatinine >177 μmol L^−1^).^11^, ^35^ The results of logistic regression analyses were presented as odds ratios (OR) with 95% confidence intervals. The threshold for statistical significance was *P*≤0.05.

### Secondary analysis

We characterised the mean HRR and the proportion of participants with impaired HRR within strata defined by RCRI. Additionally, we used univariable logistic regression to characterise the unadjusted association between RCRI-defined risk groups and impaired HRR, where the lowest-risk group was considered the reference category. We also characterised the mean HRR and the proportion of participants with impaired HRR within strata defined by NT Pro-BNP (>300 or ≤300 pg ml^−1^).^33^ To explore a potential trend in relationship between HRR and NT Pro-BNP, we plotted bar charts showing both the proportion (%) of participants with HRR≤12 beats min^−1^, and mean HRR (beats min^−1^), stratified by NT pro-BNP concentration (<100, 100–199 and ≥200 pg ml^−1^).

### Sensitivity analysis

To take account of potential confounding by heart rate-limiting medications, we repeated the primary analysis including negatively chronotropic cardiovascular medications (beta-blockers and non-dihydropyridine calcium channel antagonists) as covariates.

### Sample size calculation

This was a planned secondary analysis of a prospectively collected data. The sample size was determined based on the comparisons being made in the principal analysis, which has been published previously.^27^ For this sub-study, we included all available cases such that the sample size was based on convenience.

## Results

### Study sample

**Fig 1:**
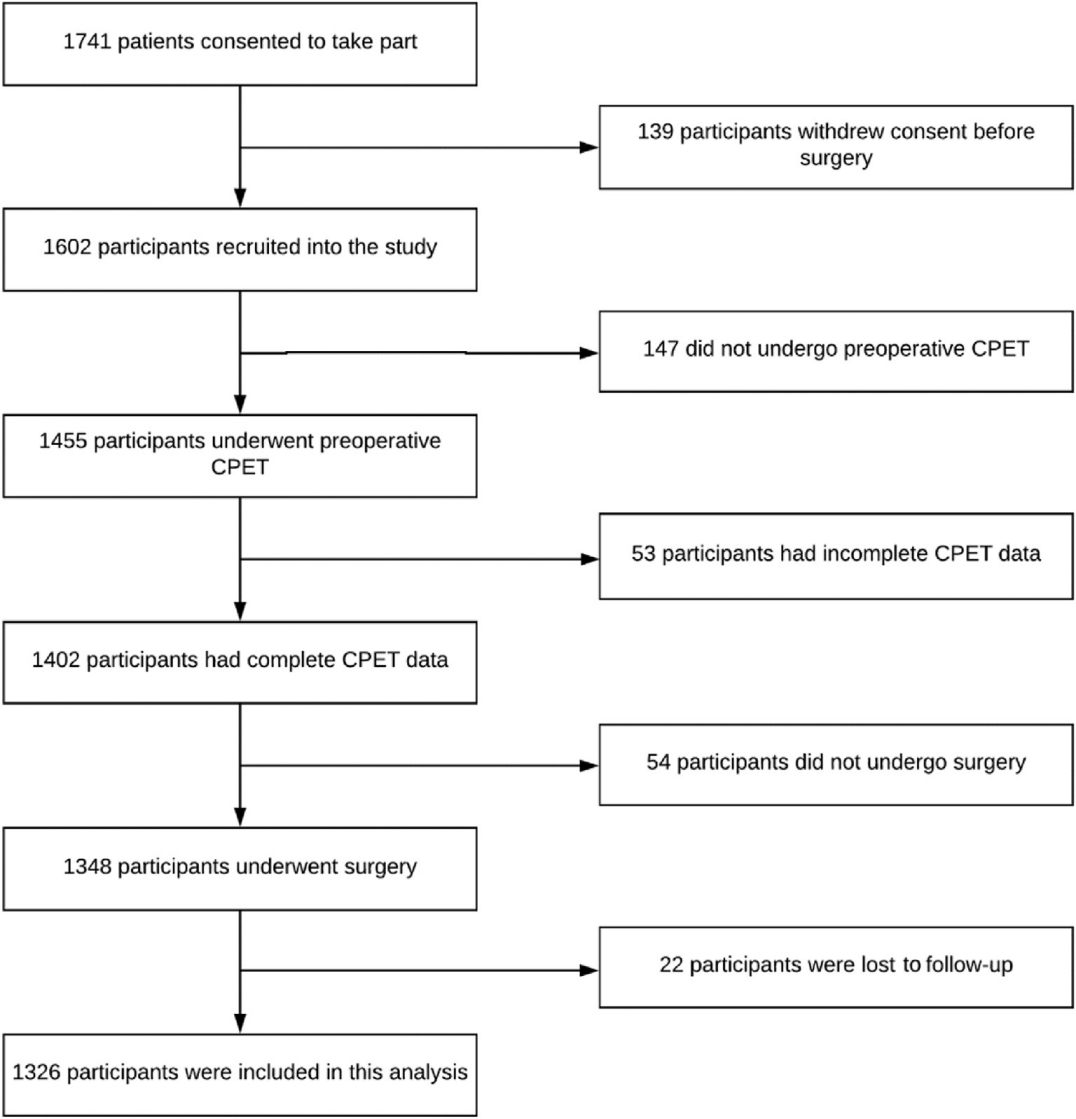
Patient flow diagram showing the number of patients included in the analysis. CPET, cardiopulmonary exercise testing.

A total of 1741 patients were recruited into the METS study between March 2013 and March 2016. After pre-defined exclusion of patients who did not undergo preoperative CPET (*n*=147) or surgery (*n*=54), or had incomplete CPET data including absent measurement of HRR (*n*=53), we analysed data obtained from 1326 participants (Fig. 1). Their mean age was 64 (10) yr; 816 (61.5%) were male and 750 (56.6%) underwent high-risk surgery. Overall, 1207 (91%) patients were classified as ASA Physical Status (ASA-PS) class 2 or 3, and 1144 (86%) underwent major abdominal, pelvic, or orthopaedic procedures. The baseline characteristics of the cohort are summarised in Table 1.

**Table 1:** Baseline patient characteristics. Descriptive data stratified by preoperative heart rate recovery (HRR) ≤12 beats min^−1^ in the first minute after the end of cardiopulmonary exercise testing. Data are presented as frequencies with percentages (%) or means with standard deviations (sd). Continuous data are reported to one decimal place and categorical data are rounded to the nearest whole number. ASA, American Society of Anesthesiologists

	Whole cohort	HRR≤12	HRR>12
Number of patients, *n*	1326	548	778
Age, mean (sd)	64.2 (10.3)	66.7 (10.0)	62.5 (10.2)
Male sex (%)	816 (61.5)	315 (57.5)	501 (64.4)
Pre-existing conditions (%)			
Atrial fibrillation	50 (3.8)	23 (4.2)	27 (3.5)
Diabetes mellitus	247 (18.6)	125 (22.8)	122 (15.7)
Hypertension	725 (54.7)	336 (61.3)	389 (50.0)
Peripheral artery disease	37 (2.8)	17 (3.1)	20 (2.6)
Chronic obstructive pulmonary disease	155 (11.7)	81 (14.8)	74 (9.5)
Surgical procedure type (%)			
Vascular	25 (1.9)	14 (2.6)	11 (1.4)
Intraperitoneal or retroperitoneal	434 (32.7)	178 (32.5)	256 (32.9)
Urological or gynaecological	398 (30.0)	161 (29.4)	237 (30.5)
Intra-thoracic	30 (2.3)	11 (2.0)	19 (2.4)
Orthopaedic	312 (23.5)	131 (23.9)	181 (23.3)
Head and neck	82 (6.2)	34 (6.2)	48 (6.2)
Other	45 (3.4)	19 (3.5)	26 (3.3)
ASA-physical status (%)			
1	99 (7.5)	33 (6.0)	66 (8.5)
2	780 (58.9)	303 (55.4)	477 (61.4)
3	427 (32.3)	203 (37.1)	224 (28.8)
4	18 (1.4)	8 (1.5)	10 (1.3)
Preoperative medication (%)			
Beta-blockers	215 (16.2)	120 (21.9)	95 (12.2)
Diltiazem or verapamil	26 (2.0)	14 (2.6)	12 (1.5)
Preoperative cardiopulmonary exercise test variables			
Resting heart rate (beats min^−1^)	77 (14.1)	81 (15.2)	75 (12.7)
Peak oxygen consumption (ml kg min^−1^)	19.3 (6.4)	17.1 (5.6)	20.8 (6.5)
Anaerobic threshold (ml kg min^−1^)	12.7 (4.1)	11.6 (3.4)	13.4 (4.4)

### Assessment of heart rate recovery

**Fig 2:**
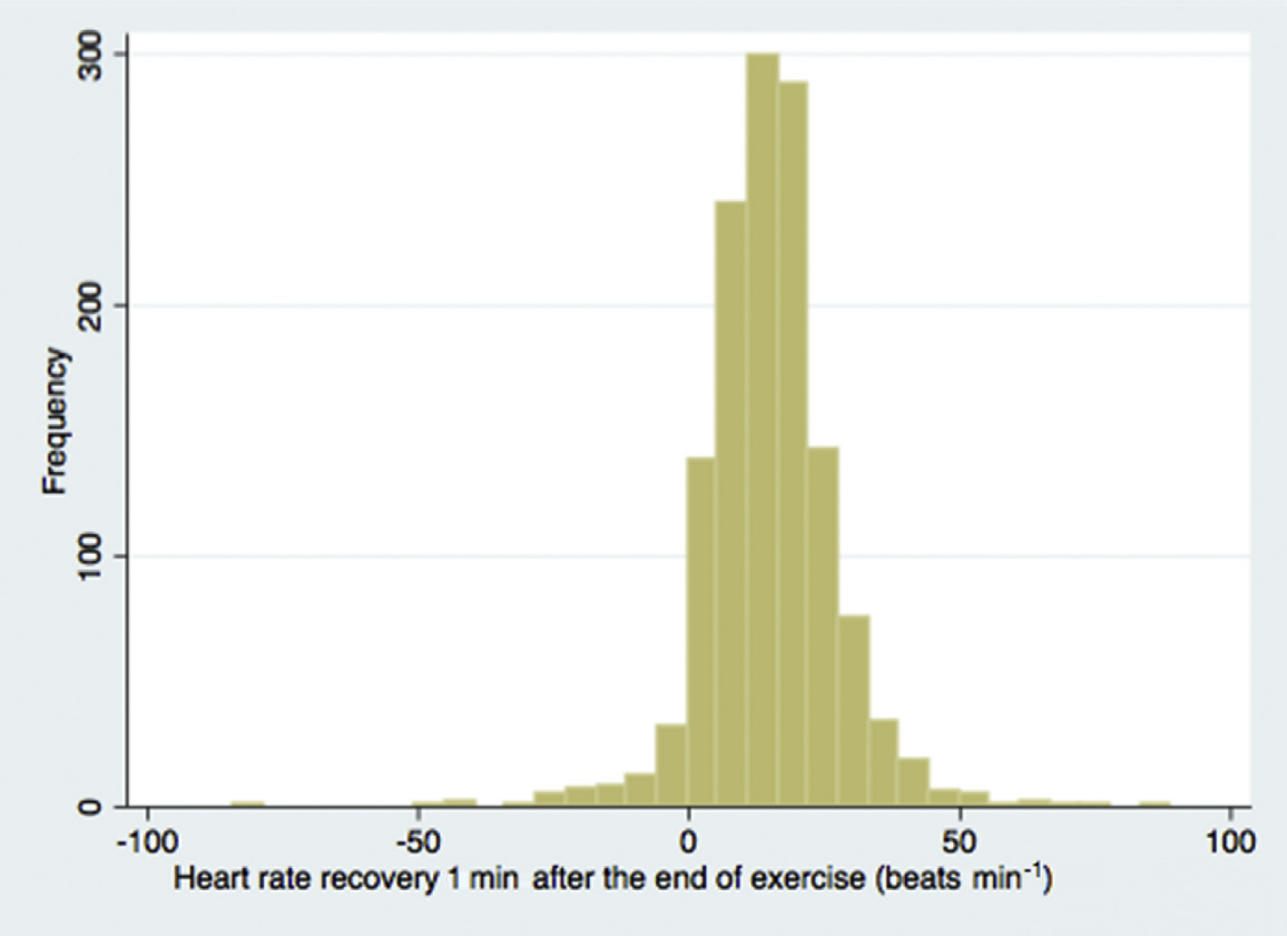
Heart rate recovery 1 min after the end of exercise. Histogram showing the frequency distribution of heart rate recovery 1 min after the end of exercise in beats min^−1^.

The mean HRR at 1 min after the end of preoperative incremental workload cardiopulmonary exercise test (HRR) was 15 (12) beats min^−1^. The distribution of HRR is shown in Fig. 2. Preoperative HRR ≤12 beats min^−1^ was present in 548/1326 (41.3%) patients. Mean resting heart rate was 77 (14) beats min^−1^. Resting heart rate, VO_2_ peak, and anaerobic threshold stratified by HRR ≤12 beats min^−1^ are shown in Table 1. Factors included in the RCRI stratified by HRR ≤12 beats min^−1^ are shown in Table 2.

**Table 2:** Revised Cardiac Risk Index (RCRI) and NT pro-BNP Risk factors included in the RCRI and RCRI score,^11^ stratified by preoperative heart rate recovery (HRR) ≤12 beats min^−1^ in the first minute after the end of cardiopulmonary exercise testing. Data are presented as frequencies with percentages (%) or median with inter-quartile range. Continuous data are reported to one decimal place and categorical data are rounded to the nearest whole number. NT Pro-BNP, N-terminal pro-hormone of brain natriuretic peptide

	Whole cohort	HRR≤12	HRR>12
Components of the RCRI (%)			
High-risk surgery	750 (56.6)	311 (56.8)	439 (56.4)
Heart failure	17 (1.3)	11 (2.0)	6 (0.8)
Coronary artery disease	153 (11.5)	84 (15.3)	69 (8.9)
Cerebrovascular disease	54 (4.1)	28 (5.1)	26 (3.3)
Preoperative creatinine >177 μmol L^−1^	100 (7.5)	43 (7.9)	57 (7.3)
Insulin therapy	54 (4.1)	26 (4.7)	28 (3.6)
RCRI score (%)			
0	419 (31.6)	167 (30.5)	252 (32.4)
1–2	871 (65.7)	355 (64.8)	516 (66.3)
≥3	36 (2.7)	26 (4.7)	10 (1.3)
NT pro-BNP (pg ml^−1^)	82 (40–166)	100 (47–222)	76 (35–137)

### Primary outcome

Postoperative myocardial injury was sustained by 168/1326 (12.7%) patients and was more frequent in patients with impaired HRR [HRR≤12: 85/548 (15.5%) patients *vs* HRR>12: 83/778 (10.7%) patients; OR, 1.54 (1.11–2.13); *P*=0.009]. When the analysis was adjusted for baseline cardiovascular risk defined by RCRI score (Supplementary Table S2) and individual component risk factors of the RCRI (Supplementary Table S3), impaired HRR remained associated with myocardial injury.

### Sensitivity analysis

When we corrected the primary analysis for heart rate-limiting medication and VO_2_ peak, the results were similar (Supplementary Table S4).

### Preoperative heart rate recovery and RCRI

**Fig 3:**
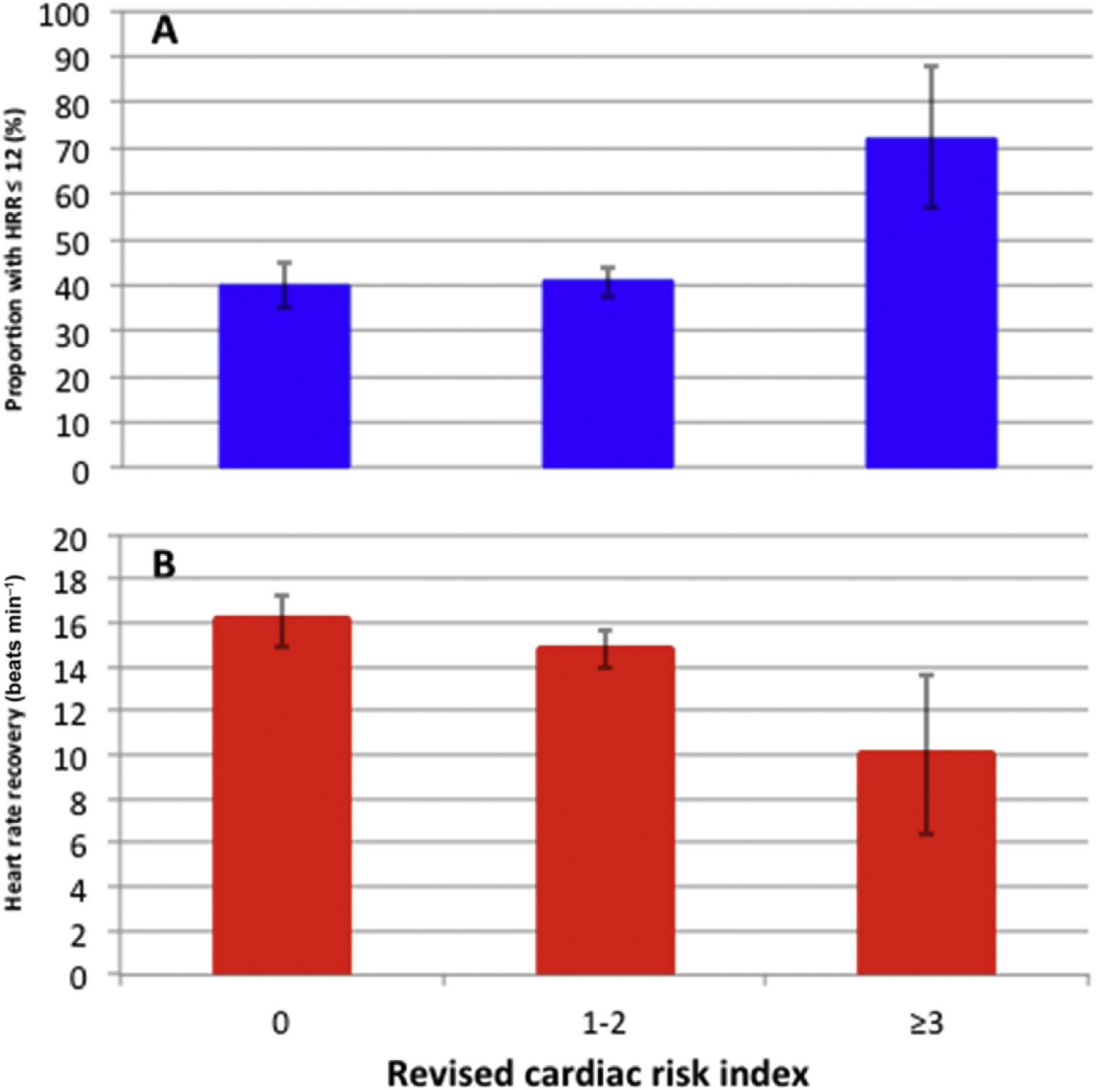
Heart rate recovery and Revised Cardiac Risk Index (RCRI). Bar charts showing (a) the proportion (%) of participants with heart rate recovery (HRR) less than or equal to 12 beats min^−1^, and (b) mean heart rate recovery (beats min^−1^), stratified by the RCRI. Error bars indicate the 95% confidence interval for the mean. Overall, 167/419 patients with RCRI=0 had heart rate recovery ≤12 beats min^−1^, 355/871 patients with RCRI=1–2 had heart rate recovery ≤12 beats min^−1^, and 26/36 patients with RCRI ≥3 had heart rate recovery ≤12 beats min^−1^. The proportion of patients with heart rate recovery ≤12 beats min^−1^ was significantly greater for RCRI ≥3 compared with the other two groups (*P*<0.01).

The RCRI is prognostically associated with cardiovascular complications after non-cardiac surgery.^11^ We found that the proportion of participants with HRR ≤12 beats min^−1^ increased with higher RCRI score (Fig. 3a). Similarly, mean HRR progressively declined in patients with increasing frequency of RCRI-defined risk factors (Fig. 3b and Supplementary Table S5). Participants with three or more RCRI-defined cardiovascular risk factors were more likely to have impaired HRR compared with those with none [RCRI≥3: 26/36 (72.2%) *vs* RCRI=0: 167/419 (39.9%); OR, 3.92 (1.84–8.34); *P*<0.001].

### Preoperative NT pro-BNP concentration

**Fig 4:**
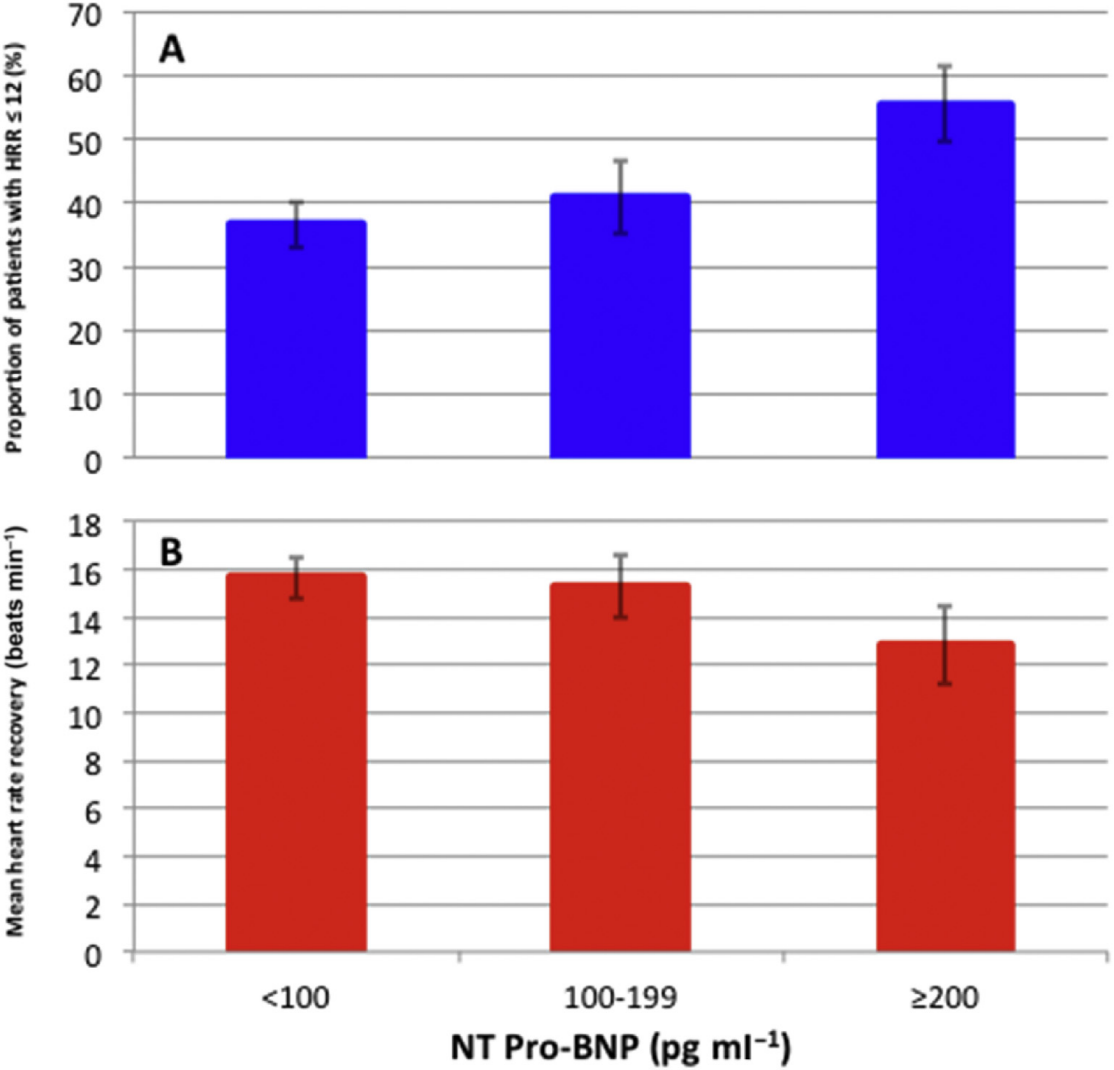
Heart rate recovery and NT Pro-BNP. Bar charts showing (a) the proportion (%) of participants with heart rate recovery ≤12 beats min^−1^, and (b) mean heart rate recovery (beats min^−1^), stratified by NT pro-BNP concentration (<100, 100–199, and ≥200 pg ml^−1^). Error bars indicate the 95% confidence interval for the mean. Overall, 263/719 (36.6%) patients with NT pro-BNP <100 pg ml^−1^ had heart rate recovery ≤12 beats min^−1^, 119/291 (40.9%) patients with NT pro-BNP 100–199 pg ml^−1^ had heart rate recovery ≤12 beats min^−1^, and 146/263 (55.5%) patients with NT pro-BNP ≥200 pg ml^−1^ had heart rate recovery ≤12 beats min^−1^. NT Pro-BNP, N-terminal pro-hormone of brain natriuretic peptide.

Elevated preoperative NT pro-BNP (>300 pg ml^−1^) is a known risk factor for postoperative cardiovascular complications.^33^, ^35^ Elevated preoperative NT pro-BNP (>300 pg ml^−1^) concentration was present in 155/1325 (12.2%) patients, of whom only 11/155 (7.1%) had a pre-existing clinical diagnosis of heart failure. Of 155 patients with elevated NT pro-BNP concentration, 96 (61.9%) had HRR ≤12 beats min^−1^ compared with 433/1119 (38.7%) with NT pro-BNP ≤300 pg ml^−1^ [OR, 2.58 (1.82–3.64); *P*<0.001]. The proportion of participants with HRR ≤12 beats min^−1^ increased with increasing concentrations of preoperative NT pro-BNP (Fig. 4a). Absolute HRR values declined in patients with increasing concentrations of preoperative NT pro-BNP (Fig. 4b).

## Discussion

The principal finding of this planned analysis of the METS study was that lower HRR after exercise, which reflects cardiac vagal dysfunction, was independently associated with myocardial injury after non-cardiac surgery after adjusting for preoperative cardiovascular risk factors (RCRI). Our study identifies an association between cardiac vagal dysfunction and objective biochemical evidence of myocardial injury after non-cardiac surgery. We used a HRR threshold that is prognostically associated with cardiovascular morbidity and mortality in large general population-based longitudinal cohorts. These present results are also consistent with our recent findings from another surgical cohort where impaired HRR before surgery was also associated with poorer postoperative clinical outcomes.^34^, ^38^

In keeping with a relationship between impaired HRR and perioperative myocardial injury, we also found that patients with three or more cardiovascular risk factors, as defined by the RCRI, had impaired HRR. This suggests that pathophysiological mechanisms other than atherosclerosis, but commonly found amongst clinical phenotypes described by the RCRI, may contribute to perioperative myocardial injury. Notably, higher RCRI scores are associated with perioperative myocardial injury irrespective of the contributing factors.^12^

We propose that preoperative risk factors, as defined by the RCRI, are pathophysiologically linked to myocardial injury, in part, through the common underlying mechanism of cardiac vagal dysfunction. Parasympathetic dysfunction is common among people with cardiometabolic risk factors that comprise the RCRI.^26^ The association between HRR and RCRI may explain why multiple factors have been repeatedly associated with perioperative myocardial injury, even though the cumulative risk is not dependent on a specific combination of risk factors incorporated into the RCRI.^6^, ^8^, ^10^, ^37^, ^39^ Consistent with these findings, we also show that elevated concentrations of plasma NT pro-BNP, which is predictive of myocardial injury mortality in non-cardiac surgery, is also associated with impaired HRR.^33^

Our data are consistent with substantial evidence supporting the hypothesis that loss of cardioprotective parasympathetic autonomic function promotes myocardial injury after non-cardiac surgery. Cardiac vagal dysfunction, as identified by low baroreflex sensitivity, reduced heart recovery after exercise or impaired heart rate variability, is common in surgical patients.^34^, ^38^, ^40^ Moreover, poor preoperative exercise performance, which is regulated by efferent vagal nerve activity,^41^ is associated with increased morbidity after major surgery.^27^ Cardiac vagal tone protects the heart through several physiological mechanisms, including inhibition of the renin–angiotensin aldosterone system and nitric oxide expression.^42^ Vagal activity may also confer an anti-inflammatory effect, which limits myocardial injury in several experimental paradigms.^22^, ^25^, ^43^ Laboratory data demonstrate that efferent vagal nerve activity reduces inflammation, via release of acetylcholine and vasoactive intestinal peptide.^44^ In humans, reduced HRR is associated with elevated neutrophil/lymphocyte ratio,^45^ a robust marker for chronic systemic inflammation that is associated with perioperative cardiovascular morbidity and mortality.^40^ Reduced cardiac vagal activity predisposes to cardiac arrhythmias,^46^ particularly atrial fibrillation, which are also associated with plasma troponin elevation.

### Study strengths and limitations

A strength of this study was its prospective, international, multi-centre design, which make the results generalisable to the majority of patients undergoing non-cardiac surgery. Secondly, the primary outcome, myocardial injury, is an objective biochemical indicator and encompasses the full spectrum of myocardial injury after non-cardiac surgery.^47^ Although HRR declines with chronological age,^26^ it is notable, yet frequently overlooked, that older age is consistently linked to perioperative myocardial injury.^6^, ^10^, ^37^ The potential for measurement error, observer bias, or both between multiple METS study sites was mitigated by the prospective use of a standardised exercise testing protocol and interpretation guidelines, and a standard case report form for collecting exercise test data.^31^ Limitations of this study include its observational design, precluding any conclusions regarding causality. These findings do not prove that impaired vagal activity is the causal mechanism behind myocardial injury; it could be a surrogate marker of the underlying mechanism. Although the use of a cut-off value of ∼12 beats min^−1^ has been demonstrated to have prognostic value in general medical populations, our ongoing work in an even larger population will refine the optimal parameter relevant for the perioperative setting.^48^, ^49^, ^50^ The addition of intraoperative haemodynamic data would add further insight into the relationship between cardiac vagal autonomic dysfunction, impaired aerobic capacity, and hypotension, which is associated with perioperative myocardial injury.^51^ Although previous studies have established that impaired HRR is strongly associated with other measures of cardiac vagal autonomic dysfunction,^34^, ^52^ the lack of other autonomic measures in this study may limit generalisability beyond CPET-derived parameters.

## Conclusions

Cardiac vagal (parasympathetic) dysfunction, characterised by impaired heart rate recovery after preoperative exercise testing, is independently associated with myocardial injury after non-cardiac surgery. These data suggest that cardiac vagal dysfunction is a plausible mechanism that contributes to perioperative myocardial injury.

## Authors’ contributions

Conception of the hypothesis: GLA.

Design of the analysis plan: all authors.

Data analysis: TEFA, GLA.

Writing paper: TEFA, RP, GLA.

Revision and critical review of the manuscript: all authors.

## Declarations of interest

The METS Study funding sources had no role in the design and conduct of the study; collection, management, analysis, and interpretation of the data; and preparation or approval of the article. RP holds research grants, and has given lectures, performed consultancy work, or both for Nestle Health Sciences, BBraun, Medtronic, GlaxoSmithKline, and Edwards Lifesciences, and is a member of the Associate editorial board of the *British Journal of Anaesthesia*; GLA is a member of the editorial advisory board for Intensive Care Medicine Experimental, is an Editor for the *British Journal of Anaesthesia*, and has undertaken consultancy work for GlaxoSmithKline; TEFA is a committee member of the Perioperative Exercise Testing and Training Society; there are no other relationships or activities that could appear to have influenced the submitted work.

## Funding

Canadian Institutes of Health Research, Heart and Stroke Foundation of Canada, Ontario Ministry of Health and Long-Term Care, Ontario Ministry of Research and Innovation, National Institute of Academic Anaesthesia, UK Clinical Research Network, Australian and New Zealand College of Anaesthetists, and Monash University grants to the METS Study. Medical Research Council and British Journal of Anaesthesia clinical research training fellowship (grant reference MR/M017974/1) to TEFA; UK National Institute for Health Research Professorship to RP; British Journal of Anaesthesia/Royal College of Anaesthetists basic science Career Development award, British Oxygen Company research chair grant in anaesthesia from the Royal College of Anaesthetists and British Heart Foundation Programme Grant (RG/14/4/30736) to GLA. Merit Awards from the Department of Anesthesia at the University of Toronto to BHC and DNW New Investigator Award from the Canadian Institutes of Health Research to DNW.
