# Supplementary material for: Cardiac vagal dysfunction and myocardial injury after non-cardiac surgery: a planned secondary analysis of the measurement of Exercise Tolerance before surgery study
Source: Br J Anaesth. 2018 Dec 17;122(2):188–97. doi: 10.1016/j.bja.2018.10.060 (PMC6354047; doi:10.1016/j.bja.2018.10.060)
Supplement: Multimedia component 1 [file mmc1.docx]

**Cardiac vagal** **dysfunction and myocardial injury after non-cardiac surgery: a planned secondary analysis of the METS study**

**SUPPLEMENTARY FILE**

**T. E. F. Abbott, PhD;^1, 2^ R. M. Pearse, MD;^1, 3,^ B.H. Cuthbertson, MD;^4, 5^ D.N. Wijeysundera, PhD;^5, 6, 7^ and G. L. Ackland, PhD;^1, 3^ for the METS study investigators.**

1. *William Harvey Research Institute, Queen Mary University of London, UK*
2. *University College London Hospital, London, UK*
3. *Barts Health NHS Trust, London, UK*
4. *Department of Critical Care Medicine, Sunnybrook Health Sciences Centre, 2075 Bayview Avenue, Toronto, Ontario, Canada*
5. *Department of Anesthesia, University of Toronto, Toronto, Ontario, Canada.*
6. *Li Ka Shing Knowledge Institute, St. Michael's Hospital, Toronto, Ontario, Canada.*
7. *Department of Anesthesia and Pain Management, Toronto General Hospital, Toronto, Ontario, Canada*

Correspondence to:

Gareth L. Ackland PhD FRCA FFICM FHEA

Translational Medicine and Therapeutics, William Harvey Research Institute

Queen Mary University of London, London EC1M 6BQ

e-mail: g.ackland@qmul.ac.uk

Tel: +44 207 882 2100

**Keywords: Observational study; heart rate; Surgery**

**INVESTIGATOR LISTING**

1. **Study Site Investigators**

**Australia –** *Alfred Hospital*: P S Myles (Site Co-Lead), M A Shulman (Site Co-Lead), S Wallace, C Farrington, B Thompson (Site CPET Lead), M Ellis, B Borg; *John Hunter Hospital*: R K Kerridge (Site Lead), J Douglas, J Brannan, J Pretto; *Nambour General Hospital*: M G Godsall (Site Co-Lead), N Beauchamp (Site Co-Lead), S Allen, A Kennedy, E Wright, J Malherbe; *Peter McCallum Cancer Centre*: H Ismail (Site Co-Lead), B Riedel (Site Co-Lead), A Melville, H Sivakumar, A Murmane, K Kenchington, Y Kirabiyik; *Prince Charles Hospital*: U Gurunathan (Site Lead), C Stonell, K Brunello, K Steele, O Tronstad, P Masel, A Dent, E Smith, A Bodger, M Abolfathi; *Princess Alexandra Hospital*: P Sivalingam (Site Co-Lead), A Hall (Site Co-Lead); *Royal Adelaide Hospital*: T W Painter (Site Co-Lead), S Macklin (Site Co-Lead), A Elliott, A M Carrera; *Royal Hobart Hospital*: N C S Terblanche (Site Lead); S Pitt, J Samuels, C Wilde; *Royal Melbourne Hospital*: K Leslie (Site Lead), A MacCormick; *Western Health*: D Bramley (Site Lead), A M Southcott, J Grant, H Taylor, S Bates, M Towns, A Tippett, F Marshall

**Canada** ***–*** *St Michael’s Hospital*: C D Mazer (Site Lead), J Kunasingam, A Yagnik, C Crescini, S Yagnik; *Sunnybrook Health Sciences Centre*: C J L McCartney (Site Co-Lead), S Choi (Site Co-Lead), P Somascanthan, K Flores; *Toronto General Hospital*: D N Wijeysundera (Site Co-Lead), W S Beattie (Site Co-Lead), K Karkouti, H A Clarke, A Jerath, S A McCluskey, M Wasowicz, J T Granton (Site CPET Lead), L Day, J Pazmino-Canizares; *Toronto Rehabilitation Institute – Rumsey Centre*: P Oh (Site Lead), R Belliard, L Lee, K Dobson; *Toronto Western Hospital*: V Chan (Site Lead), R Brull, N Ami, M Stanbrook (Site CPET Lead)

**New Zealand -** *Auckland City Hospital*: K Hagen (Site Lead), D Campbell, T Short, J Van Der Westhuizen, K Higgie, H Lindsay, R Jang, C Wong, D Mcallister, M Ali, J Kumar, E Waymouth, C Kim; *Middlemore Hospital*: J Dimech (Site Co-Lead), M Lorimer (Site Co-Lead), J Tai, R Miller, R Sara, A Collingwood, S Olliff, S Gabriel, H Houston; *Wellington Hospital*: P Dalley (Site Lead), S Hurford, A Hunt, L Andrews, L Navarra, A Jason-Smith, H Thompson, N McMillan, G Back

**United Kingdom** **–** *Aberdeen Royal Infirmary*: B L Croal (Site Lead), M Lum; *Royal Free Hospital*: D Martin (Site Lead), S James, H Filipe, M Pinto, S Kynaston; *Royal London Hospital*: R M Pearse (Site Lead), T E F Abbott, M Phull, C Beilstein, P Bodger, K Everingham, Y Hu, E Niebrzegowska, C Corriea, T Creary, M Januszewska, T Ahmad, J Whalley, R Haslop, J McNeil, A Brown, N MacDonald, M Pakats, K Greaves; *Royal Marsden Hospital*: S Jhanji (Site Co-Lead), R Raobaikady (Site Co-Lead), E Black, M Rooms, H Lawrence, M Koutra, K Pirie, M Gertsman; *Southampton General Hospital*: S Jack (Site Co-Lead), M Celinski (Site Co-Lead), D Levett, M Edwards, K Salmon, C Bolger, L Loughney, L Seaward, H Collins, B Tyrell, N Tantony, K Golder; *University College London Hospital*: G L Ackland (Site Lead), R C M Stephens, L Gallego-Paredes, A Reyes, A Gutierrez del Arroyo; *Whipps Cross Hospital*: A Raj (Site Lead), R Lifford

1. **International and National Coordinators** – B H Cuthbertson (International Co-Principal Investigator), D N Wijeysundera (International Co-Principal Investigator), R M Pearse, P S Myles, T E F Abbott, M A Shulman
2. **Central Project Office Operations Committee** – B H Cuthbertson, D N Wijeysundera, E Torres, A Ambosta, M Melo, M Mamdani, K E Thorpe, R M Pearse, T E F Abbott, P S Myles, M A Shulman, S Wallace, C Farrington, B L Croal
3. **CPET Methods Committee** – M P W Grocott, J T Granton, P Oh, B Thompson, D Levett
4. **Outcome Adjudication Committee** – G Hillis (Chair), W S Beattie, H C Wijeysundera
5. **International Steering Committee –** B H Cuthbertson (International Co-Principal Investigator), D N Wijeysundera (International Co-Principal Investigator), R M Pearse, M A Shulman, T E F Abbott, E Torres, A Ambosta, B L Croal, J T Granton, K E Thorpe, M P W Grocott, C Farrington, S Wallace, P S Myles

**Supplementary Table 1. A summary of troponin assays used at each centre. The cut-off value is the limit of the normal range, above which the participant would be considered to have myocardial injury.**

| **Assay** | **Cut-off value** | **Number of centres** |
| --- | --- | --- |
| Troponin-I Abbott Architect Immunoassay | >70 ng/L | 1 |
| Troponin-I Abbott assay | >20 ng/L | 1 |
| Troponin-I Abbott high-sensitivity assay | >25 ng/L | 2 |
| Troponin-I Abbott high-sensitivity assay | Males >25 ng/L, Females >15 ng/L | 3 |
| Troponin-I Beckman-Coulter assay | >30 ng/L | 3 |
| Troponin-I Centaur CP high-sensitivity assay | >40 ng/L | 8 |
| Troponin-T Roche high-sensitivity (STAT) assay | >14 ng/L | 4 |
| Troponin-T Roche high-sensitivity assay | >29 ng/L | 1 |
|  |  |  |

**Supplementary table 2. Impaired heart rate recovery and myocardial injury.**

Multivariable logistic regression analysis. The independent variable was impaired heart rate recovery (≤12 beats per minute within the first minute after the end of incremental exercise). The dependent variable was myocardial injury within 72 hours after the end of surgery, adjusted for Revised Cardiac Risk Index (RCRI) as an ordered categorical variable. Results are presented as adjusted odds ratios with 95% confidence intervals and associated p-values.

|  | **Myocardial Injury** | |
| --- | --- | --- |
| **Covariates** | **Odds ratio** | **p-value** |
|  |  |  |
| RCRI | - | - |
| 0 (reference) | - | - |
| 1-2 | 0.72 (0.51-1.01) | 0.058 |
| ≥3 | 1.69 (0.75-3.81) | 0.201 |
| Heart rate recovery ≤12 beats per minute | 1.50 (1.08-2.08) | 0.016 |
|  |  |  |

**Supplementary Table 3. Impaired heart rate recovery and myocardial injury: adjusted analysis.**

Multivariable logistic regression analysis. The independent variable was impaired heart rate recovery (≤12 beats per minute within the first minute after the end of incremental exercise). The dependent variable was myocardial injury within 72 hours after the end of surgery. The analysis was adjusted for individual cardiovascular risk factors in the Revised Cardiac Risk Index (RCRI), which are included as separate terms in the model. Results are presented as adjusted odds ratios with 95% confidence intervals and associated p-values.

|  | **Myocardial Injury** | |
| --- | --- | --- |
| **Covariates** | **Odds ratio** | **p-value** |
|  |  |  |
| High-risk surgery | 0.75 (0.54-1.05) | 0.091 |
| Coronary artery disease | 2.15 (1.39-3.32) | 0.001 |
| Heart failure | 0.55 (0.12-2.52) | 0.442 |
| Cerebrovascular disease | 1.50 (0.74-3.04) | 0.262 |
| Diabetes mellitus requiring insulin | 1.20 (0.56-2.55) | 0.640 |
| Preoperative creatinine >177 μmol/L | 0.65 (0.33-1.29) | 0.221 |
| Heart rate recovery ≤12 beats per minute | 1.46 (1.05-2.03) | 0.025 |
|  |  |  |

**Supplementary table 4. Impaired heart rate recovery and myocardial injury.**

**Sensitivity analysis adjusting for heart rate limiting cardiovascular medications. The independent variable was impaired heart rate recovery (≤12 beats per minute within the first minute after the end of incremental exercise). The dependent variables was myocardial injury within 72 hours after the end of surgery, adjusted for heart limiting medications and peak oxygen consumption on cardiopulmonary exercise testing. Results are presented as odds ratios with 95% confidence intervals and associated p-values.**

|  | **Myocardial Injury** | |
| --- | --- | --- |
| **Covariates** | **odds ratio** | **p-value** |
| RCRI | - | - |
| 0 (reference) | - | - |
| 1-2 | 0.70 (0.50-0.99) | 0.04 |
| ≥3 | 1.41 (0.62-3.21) | 0.42 |
| Preoperative beta-blockers | 1.62 (1.08-2.42) | 0.02 |
| Preoperative diltiazem or verapamil | 1.66 (0.61-4.51) | 0.32 |
| Peak oxygen consumption ≤ 14ml.kg.min^-1^ | 0.94 (0.62-1.42) | 0.75 |
| Heart rate recovery ≤12 beats per minute | 1.44 (1.03-2.03) | 0.03 |
|  |  |  |

**Supplementary table 5. Number of revised cardiac risk index factors stratified by heart rate recovery.**

**Heart rate recovery was considered a continuous variable, presented as mean with standard deviations. Hypothesis testing for difference in means by RCRI category using one-way ANOVA with four degrees of freedom.**

| Revised Cardiac Risk Index | Heart rate recovery (bpm) | p-value |
| --- | --- | --- |
| 0 | 16.1 (12.1) | - |
| 1 | 15.1 (11.8) | - |
| 2 | 13.0 (14.1) | - |
| 3 | 10.5 (8.9) | - |
| 4 | 7.7 (17.9) | - |
|  |  | <0.01 |
